# Iso-height axis correlates with collateral ligament balance in medial compartment knee osteoarthritis

**DOI:** 10.3389/fbioe.2025.1695631

**Published:** 2025-11-27

**Authors:** Zheng Jiang, Xiaolong Lü, Nan Zheng, Zhaoyi Fang, Yanjie Mao, Axiang He, Xuancheng Zhang, Weiming Lin, Bingbo Bao, Xianyou Zheng, Wanjun Liu

**Affiliations:** 1 Department of Orthopedics, Shanghai Sixth People’s Hospital Affiliated to Shanghai Jiao Tong University School of Medicine, Shanghai, China; 2 School of Biomedical Engineering & Med-X Research Institute, Shanghai Jiao Tong University, Shanghai, China; 3 Biodynamics Lab, Department of Orthopaedic Surgery, University of Pittsburgh, Pittsburgh, PA, United States

**Keywords:** IHA, flexion axes, soft tissue balance, MCKOA, collateral ligament

## Abstract

**Purpose:**

A dynamic iso-height Axis (IHA) was invented based on knee flexion motion and supposed to be a target to restore knee soft tissue balance, which is critical in knee surgeries. This study aims to validate the correlation between IHA and the elongation patterns of the medial and lateral collateral ligaments (MCL and LCL).

**Methods:**

Twenty-six patients with unilateral MCKOA and intact contralateral knees were enrolled. Three-dimensional models of knee were built from CT scan. The TEA, GCA and footprints of superficial, deep MCL (sMCL, dMCL) and LCL were determined based on knee surface models. Each ligament was divided into three bundle according to the anatomical feature. Patients performed stair climbing under the surveillance of dual fluoroscopic imaging system (DFIS), and the elongation of collateral ligament bundle and IHA positions of bilateral knees were calculated.

**Results:**

Compared to the native side, the sMCL and dMCL bundles were shorter (mean −2.9% and −6.2%, respectively), while the LCL medial and posterior bundles were significantly longer (mean 4.5%) in MCKOA knees. There were no significant differences in the IHA between the medial compartment knee osteoarthritis (MCKOA) knee and the native knee during daily extension. Besides, the iso-height point on the lateral femoral condyle geometrical centre axis (GCA) plane nearly overlapped with the lateral GCA point. There was a significant correlation between the differences of two-sided IHA positions and the differences of two-sided collateral ligament elongations (P < 0.01).

**Conclusion:**

This study firstly validated the correlation between IHA and the elongation patterns of the MCL and LCL, suggesting that IHA can reflect longitudinal soft tissue balance state of the knee joint. This finding provides new insights for improving design and planning in future clinical interventions.

## Introduction

The incidence of knee osteoarthritis (KOA) is increasing annually because of the ageing population and the increase in the global obesity rate (Sittisak et al., 2009). Among the different types of KOA, medial compartment KOA (MCKOA), accounting for the four-fifths of KOA cases (Wang et al., 2018) and isolating MCKOA knee tends to occur as the early stage of KOA (Bingham et al., 2006). Therefore, MCKOA is the most common form of KOA demanding knee preservation surgeries (Wagh and Razak, 2022). The collateral ligaments play a primary role in maintaining physiological laxity and stability patterns across the arc of knee flexion (Abulhasan and Grey, 2017), which exhibit distinct functional alterations with the abnormal medial-lateral gap balance of the knee joint in KOA-affected knees (Fishkin et al., 2002). Hence, there is an urgent clinical need for novel surgery-related parameters capable of quantitatively assessing collateral ligament function as the foundation for optimizing treatment strategies and rehabilitation protocols for MCKOA.

The knee joint soft tissue balance is mainly related to the collateral ligament function associated with knee joint activity. Robinson et al. (James et al., 2006) found that the superficial medial collateral ligaments (sMCL) controlled valgus at all angles and was dominant from 30° to 90° of flexion, and internal rotation in flexion, whereas the deep medial collateral ligament (dMCL) controlled tibial anterior shifts when the knee flexed and externally rotated and was a secondary restraint to valgus. In addition, the lateral collateral ligament (LCL) has been shown to play an essential role in limiting external rotation when the knee was in an extended position (Lim et al., 2012). During knee flexion-extension, the collateral ligament exhibits minimal length changes, a property referred to as “functional isometry.” (Victor et al., 2009) However, in current knee arthroplasty surgeries, this functional isometry is often compromised. Besides, the functions of collateral ligaments in the MCKOA knee still required more precise quantitative assessment. It is of clinical importance to investigate MCKOA collateral ligament functions, as such knee joint sickness might affect the physiological and kinematic properties of ligaments.

The construction of flexion axes using the anatomy and kinematics of the distal femur have been widely used to describe knee motions and guide knee surgical plans (Yu et al., 2023). Static parameters were initially employed to guide clinical practice. Transepicondylar axis (TEA) and geometrical centre axis (GCA) have been widely applied into knee joint surgery for decades. However, the surgical procedures and prostheses designed based on these axes currently faced various tricky issues, such as mid-range knee instability (Sravya et al., 2020). Current research suggests that this kind of mid-flexion instability is closely related to soft tissue imbalance caused by the surgery, especially to medial collateral ligament (MCL) (Bosco et al., 2024). Implementing dynamic parameters into clinical intervention directly was impossible pending the birth of the IHA. IHA was constructed by hundreds of calculated iso-height points on the medial and lateral femoral condyles to the tibial plane (Rao et al., 2021) and was expected to be helpful for achieving gap balance by maintaining the heights of two femoral condyles along the flexion-extension path. Previous study reported that the TEA and GCA measured varying femoral condyle heights, but the IHA resulted in minimal condyle height changes and could better represent the articulation characteristics of the knee. During flexion, the changes of the medial and lateral condyle heights were within 8.9 mm for TEA, within 4.2 mm for GCA and within 3.0 mm for IHA (Zhang et al., 2022). Although the IHA was supposed to be a target to restore knee ligament balance and has attracted attention increasingly, no studies have explored the relationship between the IHA and *in vivo* ligaments in actual clinical cases, which raised concerns when progressing further clinical research on the IHA.

The dual fluoroscopic imaging system (DFIS) technique is the most accurate non-invasive technology for *in vivo* dynamic motion tracking, with an accuracy of approximately 0.1 mm and 0.3 deg (Bingham and Li, 2006; Li et al., 2007). It has been widely used for the observation and analysis of *in vivo* knee joint kinematics (Li et al., 2009; Qi et al., 2013). Moreover, climbing stairs is one of the most demanding daily activities under weight-bearing conditions (Schwiesau et al., 2013), and such flexion-extension motion is mostly related to collateral ligament function. Therefore, DFIS offered us a viable way to identify MCL and LCL elongation patterns during staircase weight-bearing extension, which are of great clinical importance for MCKOA diagnosis and treatment.

The purposes of this study were to answer: (1) What are the positions of the flexion axes in MCKOA knees and healthy knees, respectively? Are there any characteristic differences? (2) Is there a significant difference in the elongation patterns of the collateral ligaments between MCKOA knees and healthy knees? (3) Is there a correlation between the position of the IHA and the elongation patterns of the knee collateral ligaments? (4) What are the potential clinical implications of the IHA for future applications?

## Materials and methods

### Subject information

Twenty-six patients (6 male and 20 female) who underwent unilateral MCKOA (10 left and 16 right knees) and had asymptomatic and intact contralateral extremities were recruited for our study, complying with the regulations of the Institutional Review Board (IRB No. *blinded for review*). All methods were performed in accordance with its relevant guidelines and regulations. Based on data distributions from previously published DFIS studies on quantitative knee joint kinematics (Jiang et al., 2025a), we utilized the Wilcoxon signed-rank test to determine sample size requirements. The analysis indicated that for detecting paired-sample differences with a mean deviation of 2 and variance of 2.5, at α < 0.05 and effect power >0.95, a minimum of 24 cases would be required. Besides, a post hoc effect power analysis was performed using G*Power 3.1 (Universität Kiel, Kiel, Germany), and 26 MCKOA patients included in this study had effect sizes above 0.95. The average age was 60.7 ± 3.7 years, and the average body mass index was 28.5 ± 3.6 kg/m^2^. All patients were scheduled to receive unilateral UKA because one of their knees had symptomatic varus deformity or early OA (Kellgren-Lawrence (Kellgren and Lawrence, 1957) (K-L) grade II ∼ III). The exclusion criteria were previous knee trauma, history of surgery in either lower limb, or neuropsychiatric diseases, any patellofemoral OA, KOA beyond K-L grade III or severe varus deformity.

### Experimental design

**FIGURE 1:**
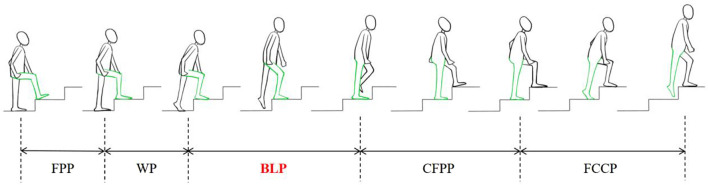
Complete gait cycle scheme during stair climbing includes a support phase [Weight-bearing Phase (WP), Body Lifting Phase (BLP), and Continuous Forward Propulsion Phase (CFPP)] and a swing phase. [Foot Contour Clearance Phase (FCCP) and Foot Placement Phase (FPP)] (Shumway-Cook and Woollacott, 2014).

Each patient underwent CT scans (Sensation 64, Siemens, Germany) for both knees with a slice thickness of 0.625 mm. The CT images were input into Amira (Thermo Fisher Scientific, Rockford IL, United States) to construct three-dimensional (3D) models of the femur and tibia. The bone coordinate systems were built according to the definition recommended by the International Society of Biomechanics (Grood and Suntay, 1983) and previous studies (Li et al., 2007; Suzuki et al., 2012). Consistent with previous studies, the stair height was set to 14 cm for climbing (Suzuki et al., 2012), which is one of the building safety standards used in China and North America (Pauls, 1991). The patients were asked to climb three stairs continuously under the surveillance of DFIS (Edge Eye, TAOIMAGE, China). The knee joint was imaged as the patients climbed the second stair using 30 pulsed snap shots per second (8 ms/pulse) by DFIS. For DFIS dynamic images, a high-frame-rate force measurement table was placed under the second step. The synchronized force signals were processed using a 5 Hz Butterworth low-pass filter (1,000 Hz, Bertec, United States), and the heel-striking to toe-off moments of the subject’s foot were extracted as the cycle of motion up the stairs (Figure 1). The body lifting phase was selected and measured in this study as a motion cycle of weight-bearing extension. The 3D model and 2D DFIS images were imported into customized software (MATLAB, 2023b; United States), adjusting the models to match the bones’ outlines on each perspective image to reconstruct the spatial position of the bones during daily extension.

The native knee was considered the reference knee with MCKOA, as the difference in knee kinematics among individuals is significantly greater than that between bilateral knees in humans. (Tsai et al., 2014). In addition, functional recovery has been increasingly adopted over mechanical axis alignment (MA) in knee corrections (Clark et al., 2023). The native sides of patients with unilateral MCKOA are less deformed, painful and more functional than their MCKOA sides, and the two sides share the same body condition, which makes native sides the most suitable references.

### Collateral ligament functions

**FIGURE 2:**
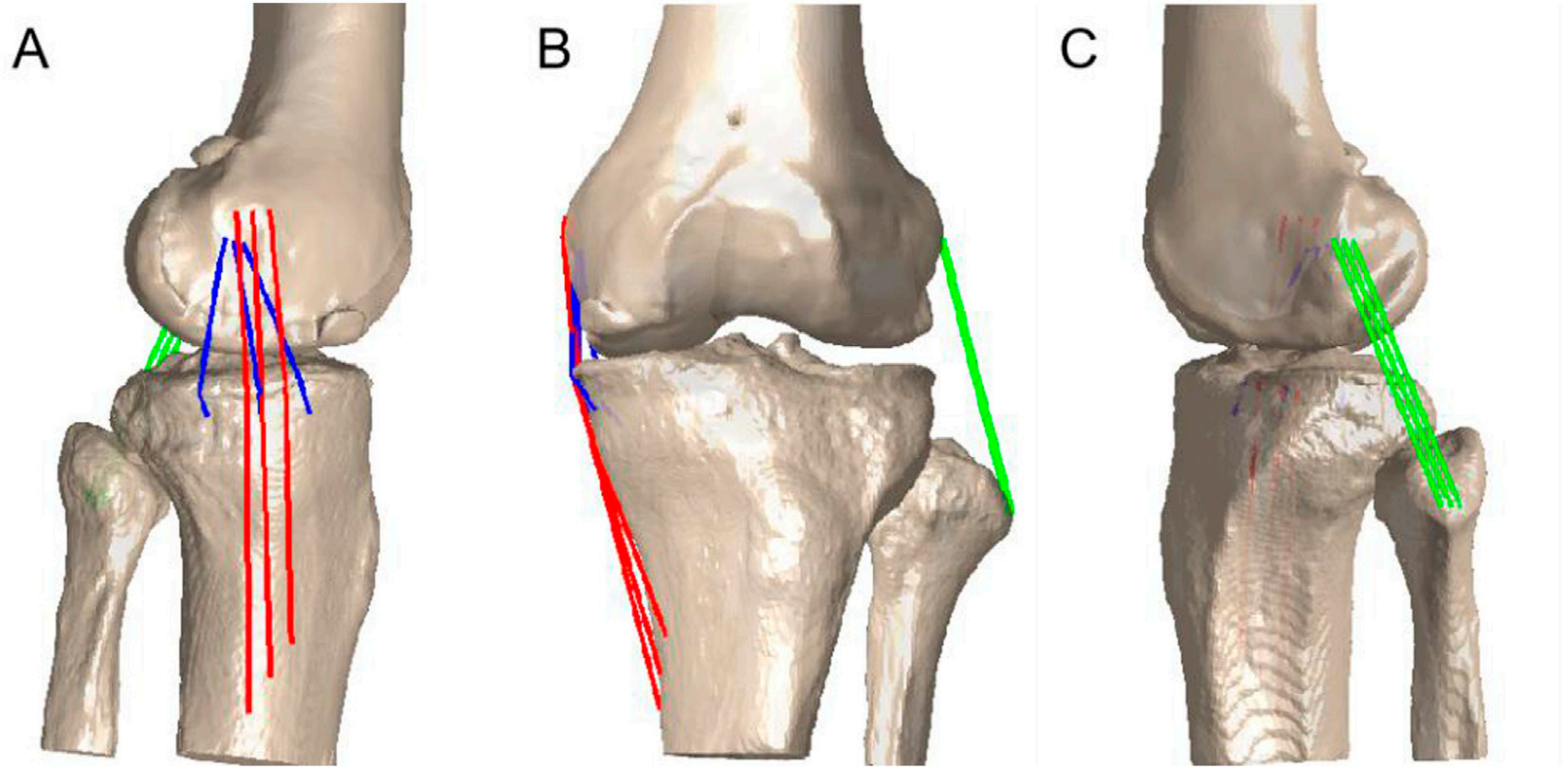
The insertion areas of the sMCL, dMCL and LCL were evenly divided into 3 equal portions to create 3 fibre bundles. The length of each bundle was measured as the distance between the centroids of the bony insertions of the bundles from the series of matched knee models, displayed on the **(A)** medial sagittal, **(C)** lateral sagittal and **(B)** frontal section planes.

To determine the length of the MCL and LCL using the shortest 3D wrapping paths (Hosseini et al., 2015), several ligament attachments on the 3D surface model of the femur, tibia, and fibula are required. This study applied the method described by Athwal et al. (Athwal et al., 2020) and LaPrade et al. (LaPrade et al., 2003) that utilized several recorded points and a two-dimensional sagittal plane coordinate system consisting of the antero-posterior dimension and proximo-distal dimension. The recorded points of the distal femur were the most anterior, posterior and distal points of the medial femoral condyle (MFC), and the horizontal distance between the most anterior and posterior ends of the femur was taken as the total length of the MFC. The recorded points of the proximal tibia were the anterior and posterior edges of the medial tibial plateau (MTP), and the horizontal distance between the most anterior and posterior ends of the tibia was taken as the total length of the MTP. The footprints of the sMCL, dMCL and LCL were subsequently calculated anatomically in relation to the knee dimensions and osseous landmarks. Afterwards, these ligament attachments were projected to the 3D bone model for further analysis and were verified by comparison with a classic anatomic study (Warren and Marshall, 1979). To minimize individual differences and examine the relative length change of the ligaments, the relative elongation of each bundle was calculated using its length during static standing as a reference, which was 100% of the ligament length (Figure 2).

### Flexion axes calculation

The DFIS technique was used to quantify the tibial 6-degrees-of-freedom (6-DOF) relative to the femur during stair climbing and static standing for the bilateral knees of each patient. Then, the femoral 6-DOF relative to the tibia were quantified during extension motion, which was described as flexion-extension (FE) coupled with internal-external (I.E.,) rotation, varus-valgus (VV) rotation and antero-posterior (AP), medio-lateral (ML), and proximo-distal (PD) translations. The TFJ translations were described in terms of the femur relative to the tibia, as weight-bearing extension is a closed-chain motion, whereas the TFJ rotations were described in terms of the tibia relative to the femur to make our findings easier to compare with those of previous studies. The motion cycle was averaged into 100 parts and was sampled at each 10% progress for analysis.

The TEA was defined by connecting the most prominent points on the medial and lateral epicondyles of the femur using the 3D femur model. To construct the GCA, two circular planes (GCA planes) fitting to the maximal cross-sections of the posterior portions of the medial and lateral condyles in the sagittal plane were established. To calculate the IHA, a series of sagittal planes was created along the mediolateral direction of the GCA (Asano et al., 2001). For each condyle, the sagittal planes were selected at distances from the GCA section plane of ±1 mm, ±4 mm, ±8 mm, ±11 mm and ±15 mm in the medial and lateral directions. To determine the iso-height points on each sagittal plane, hundreds of points were evenly distributed in the plane. A 200 × 200 point grid was sampled on the medial and lateral condylar GCA planes, with an average inter-point distance of 0.2 mm, for iso-height computation. The IH point was defined as the point within the grid that exhibited the smallest variation in distance to the tibial plateau during knee flexion-extension in stair climbing. In other words, the objective function for identifying the IH point was to minimize the variation in its distance to the tibial plateau throughout the stair-climbing cycle. All degree-of-freedom analyses in this study were normalized according to the stair-climbing cycle, a methodology consistent with our previously published work (Jiang et al., 2025b). The iso height (IH) of each point in the full range of knee flexion was calculated as the vertical distance with respect to the tibial plane (Dimitris et al., 2016). The point that had minimal changes in height with respect to the tibial plane along the knee flexion path was calculated as the iso-height point on each sagittal plane (Zhou et al., 2021). These points were subsequently used to fit a line, which was defined as the IHA.

### Statistical analysis

All measured parameters were tested for significant differences using the Wilcoxon signed-rank test. To minimize individual differences, this study conducted a correlation analysis between the IH differences and the ligament elongation differences of the bilateral knee joints. Pearson correlation analysis was used to determine the correlations between different types of data obtained in our study. A P value < 0.05 was considered to indicate a significant difference.

## Results

### Superficial MCL, deep MCL and LCL functions

During the extension of the native knee, all the lengths of collateral ligament bundles shortened at the first 20% of the motion cycle and then lengthened at 30% of the motion cycle. After that, each ligament underwent shortening again until another lengthening process at 70% of the motion cycle, followed by a mild-shortening process until the last lengthening process at the end of extension (100% of the motion cycle) (Table 1).

**TABLE 1:** Changes in the dynamic relative length of portions of the collateral ligaments of the native side during a weight-bearing extension motion cycle (Mean ± SD).

		Motion cycle									
Ligaments	Bundles	0%	10%	20%	30%	40%	60%	70%	80%	90%	100%
sMCL	AP (%)	103.63 ± 4.22	99.68 ± 3.63	96.65 ± 3.23	102.99 ± 10.39	96.69 ± 8.87	90.1 ± 7.38	97.26 ± 5.99	94.45 ± 6.08	91.68 ± 6.1	103.63 ± 4.22
	MP (%)	103.58 ± 4.18	99.79 ± 3.65	96.9 ± 3.27	102.5 ± 10.26	96.64 ± 8.98	90.34 ± 7.24	97.27 ± 6.16	94.59 ± 6.21	91.95 ± 6.19	103.58 ± 4.18
	PP (%)	103.47 ± 3.96	99.93 ± 3.48	97.22 ± 3.17	101.95 ± 9.7	96.64 ± 8.7	90.73 ± 7.13	97.17 ± 5.8	94.64 ± 5.83	92.15 ± 5.79	103.47 ± 3.96
dMCL	AP (%)	103.26 ± 3.72	100.02 ± 3.31	97.5 ± 3.05	101.41 ± 9.15	96.71 ± 8.43	91.23 ± 6.99	96.99 ± 5.64	94.63 ± 5.63	92.3 ± 5.55	103.26 ± 3.72
	MP (%)	102.9 ± 3.39	99.99 ± 3.08	97.69 ± 2.91	100.65 ± 8.44	96.65 ± 7.98	91.73 ± 6.8	96.69 ± 5.82	94.52 ± 5.77	92.37 ± 5.64	102.9 ± 3.39
	PP (%)	102.75 ± 3.34	100.17 ± 3.08	98.07 ± 2.88	100.48 ± 8.49	97.17 ± 7.97	92.77 ± 6.75	96.27 ± 6.06	94.3 ± 5.99	92.35 ± 5.84	102.75 ± 3.34
LCL	AP (%)	102.46 ± 3.38	100.18 ± 3.15	98.32 ± 2.98	100.11 ± 8.7	97.4 ± 8.27	93.54 ± 7.07	96.11 ± 6.37	94.34 ± 6.3	92.6 ± 6.14	102.46 ± 3.38
	MP (%)	102.21 ± 3.37	100.17 ± 3.16	98.53 ± 3.02	99.88 ± 8.76	97.5 ± 8.43	93.97 ± 7.15	96.04 ± 6.79	94.45 ± 6.69	92.89 ± 6.51	102.21 ± 3.37
	PP (%)	102.01 ± 3.35	100.23 ± 3.19	98.77 ± 3.03	99.86 ± 8.78	97.83 ± 8.5	94.56 ± 7.11	96.03 ± 7.11	94.61 ± 7.01	93.23 ± 6.81	102.01 ± 3.35

AP, anterior portion; MP, middle portion; PP, posterior portion.

On the MCKOA side, all three collateral ligaments underwent a process of shortening first. Only the anterior and medial bundles of the sMCL lengthened slightly at the 30% of the motion cycle. Afterwards, all the ligament bundles continued to shorten until lengthening at 70% of the motion cycle, followed by a mild shortening process until the last lengthening stage at the end of extension. During extension, the amplitude of the fluctuations in the length changes in MCKOA collateral ligaments is more pronounced compared to the native side (Table 2).

**TABLE 2:** Dynamic relative length changes in MCKOA-side collateral ligament portions during a weight-bearing extension motion cycle (Mean ± SD).

Ligaments	Bundles	0%	10%	20%	30%	40%	60%	70%	80%	90%	100%
sMCL	AP (%)	100.12 ± 3.97	96.42 ± 3.3	93.69 ± 2.79	94.67 ± 9.77	89.68 ± 8.65	85.18 ± 8.28	100.63 ± 4.3	97.87 ± 4.15	94.83 ± 4.16	100.12 ± 3.97
	MP (%)	100.16 ± 4.03	96.64 ± 3.38	94 ± 2.84	94.22 ± 9.83	89.81 ± 8.99	85.88 ± 8.34	100.79 ± 4.16	98.17 ± 4	95.25 ± 3.95	100.16 ± 4.03
	PP (%)	100.16 ± 3.93	96.9 ± 3.3	94.49 ± 2.81	94.1 ± 9.45	90.17 ± 8.76	86.57 ± 8.29	100.98 ± 4.25	98.51 ± 4.12	95.76 ± 4.1	100.16 ± 3.93
dMCL	AP (%)	100.08 ± 3.82	97.1 ± 3.29	94.87 ± 2.89	93.87 ± 9.17	90.38 ± 8.75	87.15 ± 8.21	101.19 ± 4.62	98.89 ± 4.49	96.31 ± 4.45	100.08 ± 3.82
	MP (%)	99.88 ± 3.82	97.21 ± 3.28	95.19 ± 2.89	93.51 ± 9.13	90.6 ± 8.46	87.78 ± 7.86	101.2 ± 4.91	99.09 ± 4.79	96.69 ± 4.73	99.88 ± 3.82
	PP (%)	99.59 ± 3.69	97.2 ± 3.19	95.38 ± 2.83	92.98 ± 9.02	90.56 ± 8.19	88.19 ± 7.59	101.14 ± 5.2	99.22 ± 5.1	97.01 ± 5.01	99.59 ± 3.69
LCL	AP (%)	99.4 ± 3.57	97.23 ± 3.11	95.59 ± 2.77	92.72 ± 8.89	90.78 ± 7.98	88.77 ± 7.31	101.07 ± 5.35	99.31 ± 5.28	97.29 ± 5.19	99.4 ± 3.57
	MP (%)	99.32 ± 3.49	97.33 ± 3.06	95.88 ± 2.71	92.76 ± 8.72	91.21 ± 7.67	89.36 ± 7.08	101.01 ± 5.67	99.4 ± 5.62	97.54 ± 5.55	99.32 ± 3.49
	PP (%)	99.18 ± 3.58	97.4 ± 3.1	96.09 ± 2.74	92.74 ± 8.79	91.44 ± 7.63	89.99 ± 6.68	101.07 ± 6.14	99.61 ± 6.1	97.9 ± 6.01	99.18 ± 3.58

AP, anterior portion; MP:, middle portion; PP, posterior portion.

**FIGURE 3:**
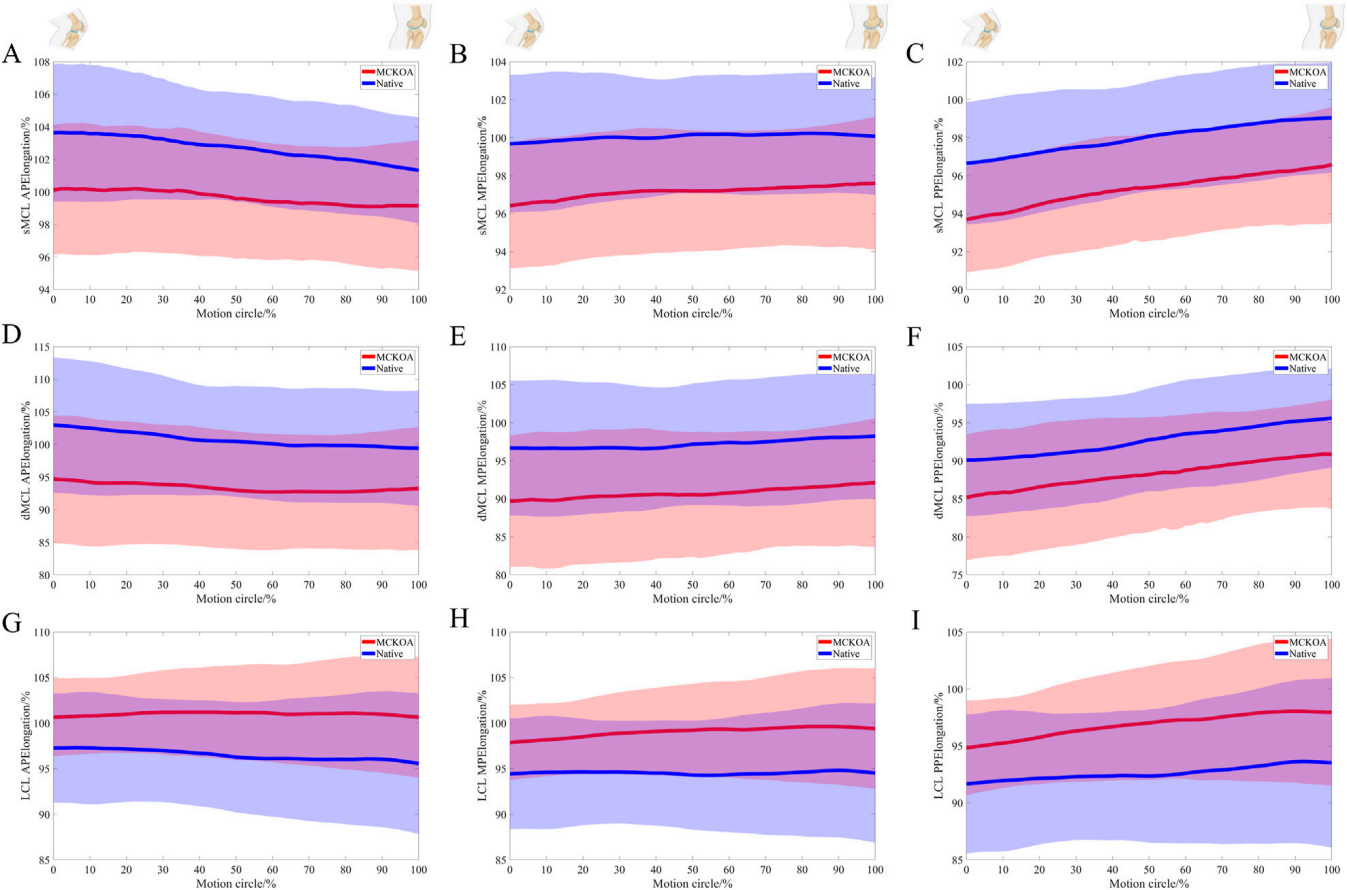
The relative length changes in the anterior **(A)**, medial **(B)**, and posterior **(C)** portions of the sMCL; the anterior **(D)**, medial **(E)**, and posterior **(F)** portions of the dMCL; and the anterior **(G)**, medial **(H)**, and posterior **(I)** portions of the LCL during the stair climbing motion between the two extremities during extension.

When comparing the elongation between the MCKOA side and the native side, both the sMCL (mean −2.9%) and dMCL (mean −6.2%) were shorter in the MCKOA knees than in the native knees. At approximately 30%–60% of the motion cycle, the medial and posterior portions of the LCL (mean 4.5%) were significantly longer on the MCKOA side than on the native side (Table 3; Figure 3).

**TABLE 3:** Elongation differences in 3 collateral ligament portions between the 2 sides during the motion cycle of weight-bearing extension (mean ± SD).

Ligaments	Bundles	0%	10%	20%	30%	40%	60%	70%	80%	90%	100%
sMCL	AP (%)	−3.51 ± 5.39^*^	−3.42 ± 5.63^*^	−3.31 ± 5.6^*^	−3.18 ± 5.61^*^	−3.02 ± 5.57^*^	−3.06 ± 5.84^*^	−2.89 ± 5.9^*^	−2.83 ± 5.96^*^	−2.58 ± 6.13^*^	−2.16 ± 6.49
	MP (%)	−3.26 ± 4.63^*^	−3.15 ± 4.82^*^	−3.03 ± 4.8^*^	−2.92 ± 4.87^*^	−2.78 ± 4.87^*^	−2.96 ± 5.22^*^	−2.84 ± 5.31^*^	−2.82 ± 5.41^*^	−2.68 ± 5.49^*^	−2.48 ± 5.86^*^
	PP (%)	−2.96 ± 3.89^*^	−2.9 ± 4.07^*^	−2.74 ± 4.06^*^	−2.62 ± 4.21^*^	−2.51 ± 4.23^*^	−2.74 ± 4.56^*^	−2.65 ± 4.69^*^	−2.68 ± 4.78^*^	−2.67 ± 4.9^*^	−2.47 ± 5.23^*^
dMCL	AP (%)	−8.32 ± 14.14^*^	−8.28 ± 14.55^*^	−7.85 ± 14.4^*^	−7.54 ± 14.49^*^	−7.14 ± 14.47^*^	−7.39 ± 15.39^*^	−7.12 ± 15.62^*^	−7.12 ± 15.76^*^	−6.67 ± 15.91^*^	−6.18 ± 16.79
	MP (%)	−7.01 ± 10.54^*^	−6.83 ± 11.43^*^	−6.46 ± 11.28^*^	−6.33 ± 11.57^*^	−6.05 ± 11.55^*^	−6.62 ± 12.43	−6.28 ± 12.63^*^	−6.39 ± 12.87^*^	−6.35 ± 13.23^*^	−6.11 ± 14.16^*^
	PP (%)	−4.92 ± 8.64^*^	−4.46 ± 8.99^*^	−4.16 ± 8.55^*^	−4.07 ± 8.43^*^	−3.95 ± 8.04^*^	−4.77 ± 8.07^*^	−4.61 ± 8.04^*^	−4.57 ± 7.88^*^	−4.68 ± 8.02^*^	−4.76 ± 8.96^*^
LCL	AP (%)	3.37 ± 8.01	3.52 ± 8.41	3.81 ± 8.79	4.2 ± 9.3	4.51 ± 9.91	4.96 ± 11.02	4.97 ± 11.77	5.05 ± 12.55	4.93 ± 13.22	5.07 ± 13.76
	MP (%)	3.42 ± 7.92	3.57 ± 8.27	3.87 ± 8.65	4.26 ± 9.16^*^	4.57 ± 9.74^*^	4.97 ± 10.85^*^	4.96 ± 11.6	4.99 ± 12.42	4.8 ± 13.11	4.88 ± 13.66
	PP (%)	3.15 ± 7.65	3.3 ± 7.93	3.6 ± 8.34	4.01 ± 8.85	4.32 ± 9.4^*^	4.69 ± 10.44^*^	4.65 ± 11.19	4.67 ± 12.03	4.43 ± 12.71	4.43 ± 13.25

AP, anterior portion; MP, middle portion; PP, posterior portion. * Indicates a statistically significant difference (p value < 0.05).

### Comparison of flexion axes’ locations

**FIGURE 4:**
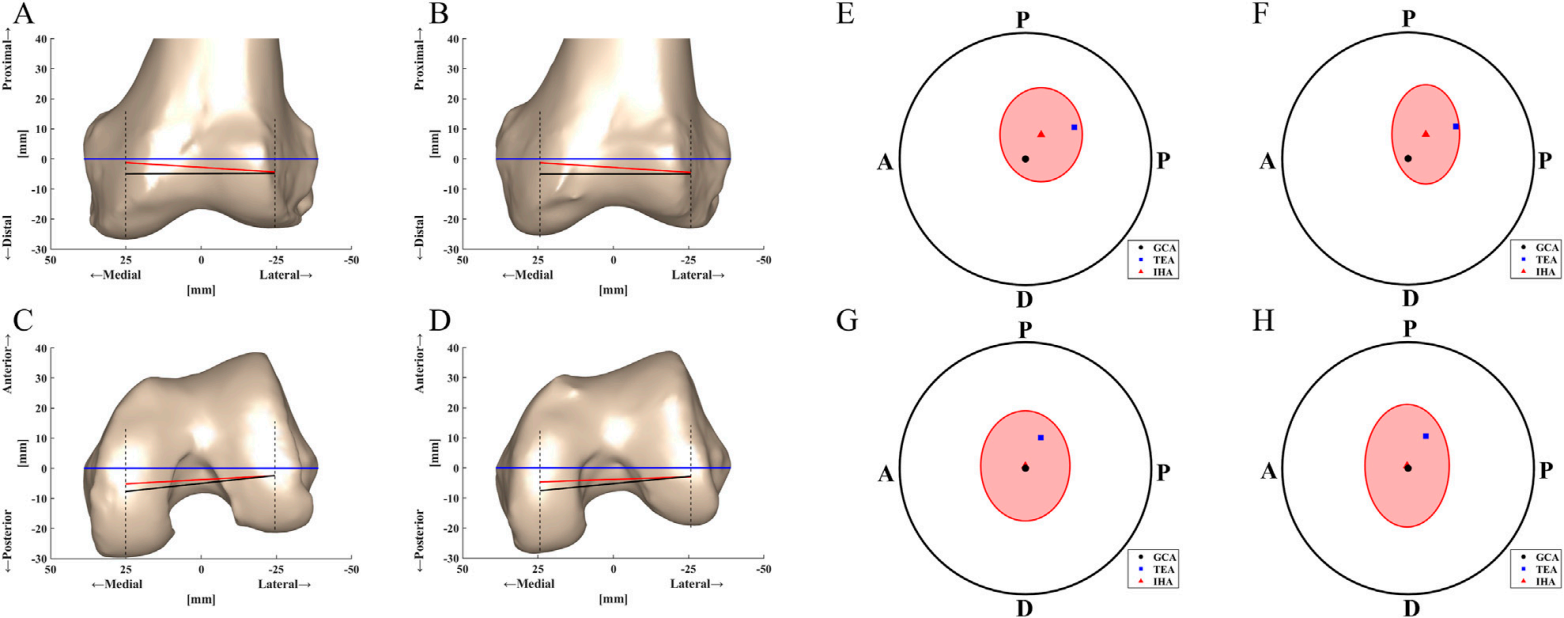
TEA, GCA and IHA locations displayed on the different planes of two-sided knees with their standard deviation projections depicted on different two-sided GCA planes: **(A)** MCKOA frontal section plane, **(B)** Native frontal section plane, **(C)** MCKOA transverse section plane, **(D)** Native transverse section plane, **(E)** MCKOA side knee medial condyle GCA plane, **(G)** MCKOA side knee lateral condyle GCA plane, **(F)** Native side knee medial condyle GCA plane and **(H)** Native side knee lateral condyle GCA plane. A–P: Anterior-Posterior; P–D: Proximal-Distal.

The differences in the sagittal projections of the TEA, GCA and IHA locations on each femoral condyle GCA plane were significant for both the MCKOA and native knees. The TEA was located more proximally and anteriorly than the GCA, especially on the medial condyle GCA plane. On the MCKOA side, the IHA locations projected on the medial condyle GCA plane were more proximal (mean 3.8 mm) than those on the GCA plane and more posterior (mean 5.2 mm) than those on the TEA. On the lateral condyle GCA plane, the IHA location projection was not significantly different from that of the TEA. On the native side, the IHA locations projected to the medial condyle were more proximal (mean 3.8 mm) and anterior (mean 2.8 mm) than those of the GCA and more posterior (mean 4.7 mm) than those of the TEA (Table 4; Figure 4).

**TABLE 4:** Differences in TEA, GCA and IHA projections on each femoral condyle GCA plane between native and MCKOA knees (mean ± SD).

	Flexion axes	Medial-PD	Medial-AP	Lateral-PD	Lateral-AP
MCKOA side	GCA	0.0	0.0	0.0	0.0
	TEA	4.9 ± 2.3^a^	7.6 ± 2.2^a^	4.8 ± 2.1^a^	2.4 ± 1.4^a^
	IHA	3.8 ± 7.3^b^	2.4 ± 6.4^b^ ^$^	0.4 ± 8.5	0 ± 6.9
Two sides TEA differences (P value)		0.61	0.50	0.64	0.31
Two sides IHA differences (P value)		0.36	0.26	0.60	0.60
Native side	GCA	0.0	0.0	0.0	0.0
	TEA	5 ± 2.2^a^	7.5 ± 2.7^a^	4.9 ± 1.7^a^	2.7 ± 1.6^a^
	IHA	3.8 ± 7.8^b^	2.8 ± 5.3^b^ ^c^	0.4 ± 9.4	−0.2 ± 6.5

The data are presented as the mean values and their corresponding standard deviations (the GCA, axis is the baseline of the other 2 axes, and positive values indicate anterior or proximal translations on the GCA, axis).

^a^ Indicates a statistically significant difference in mean values between TEA, and GCA, projections.

^b^ Indicates a statistically significant difference in mean values between GCA, and IHA, projections.

^c^ Indicates a statistically significant difference in mean values between TEA, and IHA, projections.

There were no statistically significant differences in the TEA or IHA position projections between the two sides.

### The correlation between two-sided sMCL, dMCL, and LCL elongation differences and two-sided IH differences

**FIGURE 5:**
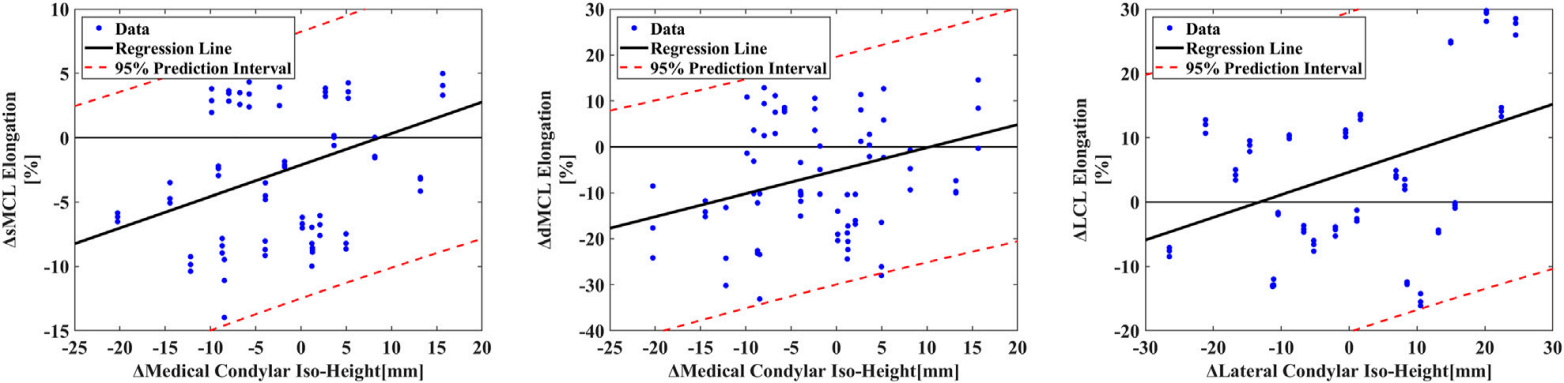
The correlation between the distance of iso-height points on two-sided femoral condyle GCA planes to the tibial plane and two-sided sMCL elongation, dMCL elongation and LCL elongation during extension.

After Pearson correlation analysis, correlations between two-sided sMCL (R_1_ = 0.44) and dMCL (R_2_ = 0.39) elongation differences and two-sided IH differences on the medial GCA plane were found (P < 0.001). The calculated relationship is displayed in Equations 1, 2. Similarly, the correlation Equation 3 between the two-sided LCL elongation difference and the two-sided IH difference on the lateral GCA plane was calculated (R_3_ = 0.35, P < 0.01). The 95% confidence intervals for each group’s R values are as follows: R_1_-value [0.24–0.60], R_2_ [0.18–0.56], R_3_ [0.14–0.53]. Units for all equations: ΔIH (mm), Δelongation (%) (Figure 5).

In addition, there was no correlation between two-sided sMCL, dMCL, or LCL elongation differences and two-sided TEA or GCA location differences after our systemic analysis. (P > 0.05)

△sMCL elongation=0.24×△IH−2.12 (1)

△dMCL elongation=0.5×△IH−5.17 (2)

△LCL elongation=0.35×△IH+4.65 (3)

## Discussion

Our study was the first to apply collateral ligament elongations and flexion axes analyses to the motion pattern study of the MCKOA knee, which provided a new perspective for understanding the mediolateral balance of the knee joint. During extension, shorter MCL and longer LCL were found in MCKOA knee compared with its native side. Furthermore, the IHA position correlated with collateral ligament elongation, which can be applied to quantify soft tissue balance.

Primary knee stabilization is achieved through knee ligaments, and the characteristic collateral ligament elongations in the MCKOA knee may have great clinical significance. The knee is reinforced by the MCL and LCL, which prevent excessive varus and valgus displacement of the tibia in relation to the femur (Abulhasan and Grey, 2017). Previously, Sang Eun Park et al. (Sang Eun et al., 2006) reported that collateral ligaments might be loaded non-uniformly throughout knee flexion, with the length of some regions increasing with flexion, whereas that of other regions decreased. They detected large variation in the bundle lengths of the dMCL throughout the flexion path of the knee, indicating that the dMCL might play an important role during normal knee flexion. In our study, this finding was further refined and quantified. The elongation pattern of the MCL and LCL were similar to a wave with 2 peaks (at 30% and 70% of the motion cycle) during knee extension. The highest peak value occurs at 30% of the motion cycle on the native side, whereas on the MCKOA side, the highest peak value is observed at 70% of the motion cycle. This finding might serve as a novel approach for preoperative assessment of knee ligament function in MCKOA patients. Furthermore, compared with native side, the sMCL (mean −2.9%) and dMCL (mean −6.2%) of the MCKOA side were significantly shorter, whereas the LCL (mean 4.5%) appeared significantly longer, which was consistent with the extra varus rotation of the MCKOA tibia found in previous 6-DOF analysis (Hoshi et al., 2016). Besides, Xia et al. (Chunjie et al., 2024) described the *in vivo* elongation patterns of the MCL and LCL after UKA, where the correlation between dMCL elongation and the joint space for UKA during middle and deep flexion was reported. Likewise, the differential elongation patterns in collateral ligaments between MCKOA and normal knees can reflect the joint space condition in MCKOA correspondingly. However, whether such collateral ligaments and joint space characteristics of MCKOA indicate significant knee joint imbalance and require clinical intervention may be elucidated by the analysis of the IHA.

Our study firstly applied IHA analysis into the comparison between the MCKOA and native knees. Previous study reported that the angles of the IHA with the GCA and the TEA were significantly different in the frontal and transverse planes (Yu et al., 2023) and Rao et al. (Rao et al., 2021) observed weight-bearing condyle motion in the knee and reported that the IHA was posterior and distal to the TEA and anterior to the GCA, which was consistent with our findings. However, differed from the findings of IHA observed in severe KOA, our study revealed that there were no significant differences in the IHA between the MCKOA knee and the native knee during extension, suggesting that IHA may remain relatively stable during mid-range, weight-bearing activities in MCKOA. However, whether this can justify single-limb surgical planning requires confirmation through an equivalence design or a larger sample. Interestingly, the iso-height point on the lateral femoral condyle GCA plane nearly overlapped with the lateral GCA point in our observation. A possible explanation for this might be that the morphology of the lateral tibial plateau is relatively flat and that the posterior portion of the lateral condyle approximates a sphere, which is close to a ball rolling on a plane. During daily staircase motion, a middle-amplitude flexion movement, excessive rotation of the femur does not occur. Therefore, such an ideal model has been widely used in lateral UKA implant designs, such as Oxford knee (Smith et al., 2020). However, the iso-height point on the medial femoral condyle GCA plane differed from the medial GCA point significantly, which indicated that the above-mentioned ideal model might demand relevant adjustments for guiding medial UKA regarding collateral ligament functions.

This study validates a clinically relevant correlation of significant practical value. Significant correlations were found between the differences in collateral ligament elongation patterns and the differences in vertical distance to the tibial plane of the iso-height points on the GCA planes, i.e., the collateral ligaments lengthened with increasing IH. Although the correlation between the IH and collateral ligament function is not particularly significant, this is likely because longitudinal soft tissue balance of the knee joint involves not only the collateral ligaments but also other ligamentous and soft tissue structures. Therefore, the clinical application of IH aims to achieve comprehensive longitudinal soft tissue balance during knee flexion (Hosseini et al., 2015). Besides, these correlation equations might be useful when further planning knee surgery. These findings confirmed that the IHA could function as a new flexion axis to guide knee surgical treatments and related implant designs with the advantage of reflecting the collateral ligament balance.

Contrary to total knee arthroplasty (TKA), knee preservation surgery like unicompartmental knee arthroplasty (UKA) does not involve intraoperative soft-tissue release commonly. Therefore, the restoration of soft tissue balance in UKA mainly relies on surgical technique or component design. Previous studies reported that maintaining soft tissue balance in knee joint surgery is of significant value for accelerating postoperative rehabilitation and preventing the revision related to malalignment and soft tissue imbalance (Kumar and Dorr, 1997; Kenneth et al., 2017). However, the complex and diverse functions of knee ligaments make precise assessment and restoration of soft-tissue balance challenging (Sharkey et al., 2002). Such imbalance was related to the failure of UKA (Matsuzaki et al., 2017). Since a new theory named natural balanced alignment (NBA), which was guided by a new flexion axis named the iso-height axis (IHA), was proposed, it has been increasingly acknowledged in achieving knee joint soft tissue balance and gap balance in knee surgical treatments (Rao et al., 2021; Zhang et al., 2022). To be more specific, a critical challenge remains in determining how to determine the optimal osteotomy strategy for knee gap balance, which can restore knee joint soft tissue balance and improve patient satisfaction. Our findings confirmed the relationship between the IHA and collateral ligament elongation patterns, which may provide a reference for conventional, computer-assisted navigation and robotic surgery planning in various knee preservation surgical procedures and related component designs. For example, kinematic data can be collected from patients while walking on level ground, climbing stairs, and performing squats preoperatively. Then, the IHA of such three movements are calculated separately. Based on the distinct flexion angles required for these daily activities, different components of the UKA prosthesis are customized accordingly, ultimately resulting in a personalized implant that meets the patient’s functional needs for daily movements. Further clinical studies are required to validate this approach.

The limitations of this study should be acknowledged. The study focused only on the extension phase in daily stair-climbing movement, which is a type of middle-amplitude extension movement. Other knee joint movements, such as running, walking, and squatting, should be measured in further studies. In addition, the native knees of these patients may not be in perfect health, either because there are no clinical symptoms or because the varus deformity is mild enough to avoid surgery. Therefore, adding a group of normal volunteers might be helpful for making our observations more comprehensive. Finally, The identification of the IHA for each affected knee is still complex due to the limited availability of DFIS. However, the application of artificial intelligence to develop a big data regression model may simplify this process. Despite these limitations, this study demonstrated the collateral ligament elongation patterns and IHA position of the MCKOA knees and relatively healthy knees under the same body conditions, which provides a reference for improving knee preservation surgeries.

## Conclusion

Our findings have implications for clarifying the flexion axes and MCL and LCL elongation patterns of the MCKOA knee, together with the relationship between the IHA and collateral ligament elongation patterns. Our study detected the different elongation patterns between MCKOA knee and its native side, together with the shortness of the MCL and lengthening of the LCL in the MCKOA knee. In addition, the correlations between two-sided IHA and collateral ligament elongation differences were found, confirming the priority of IHA in reflecting *in vivo* soft tissue balance.

## Data Availability

The raw data supporting the conclusions of this article will be made available by the authors, without undue reservation.
